# Subcutaneous Nodules Caused by *Tropheryma whipplei* Infection

**DOI:** 10.3201/eid2803.211989

**Published:** 2022-03

**Authors:** Lili Wang, Peng Su, Li Song, Lintao Sai

**Affiliations:** Shandong University Qilu Hospital, Shandong, China

**Keywords:** Whipple disease, subcutaneous nodules, skin manifestations, Tropheryma whipplei, bacteria, infection

## Abstract

To help clarify the clinical manifestations, diagnosis, and treatment for Whipple disease, we report a case of a man in China infected with *Tropheryma whipplei.* The patient had multiple subcutaneous nodules as the only manifestation, which was not consistent with the typical symptoms of *T. whipplei* infection.

Whipple disease was reported in 1907 and is a chronic infectious disease caused by the bacterium *Tropheryma whipplei* (*1*). This disease can involve multiple organs and systems and has a variety of clinical manifestations, in which arthralgia and digestive disorders are the most common first symptoms. Because of the variety and confusion of symptoms, the average time to diagnosis is >6 years (*2*). We report an elderly man given a diagnosis of *T. whipplei* skin infection and aim to increase awareness of diagnosis and treatment for Whipple disease.

A 62-year-old man (farmer) came to a dermatology clinic in Shandong, China, during November 2020 because of multiple subcutaneous nodules. The patient was otherwise healthy. These nodules were the only manifestation; the patient had no fever, arthralgia, diarrhea, malabsorption, or emaciation. The subcutaneous nodules first appeared on the left waist during January 2020. Nodules were ≈0.3 cm × 0.3 cm and gradually increased to ≈2.0 cm × 2.0 cm before treatment. Nodules then appeared successively on the right axilla, back, left thigh, and waist. These subcutaneous nodules were 0.8 cm × 2.5 cm, firm, and painless. The surfaces of 2 nodules on the left thigh were ulcerated. 

The nodules were removed surgically at a local hospital during March and August 2020 (Figure, panel A). However, pathologic examination was not performed, and treatment was not given.

**Figure:**
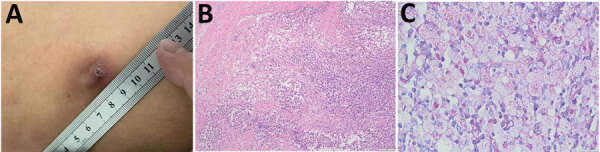
Analysis of subcutaneous nodules caused by *Tropheryma whipplei* infection in a patient in China, 2020. A) Surface of subcutaneous nodule on the left thigh showing ulceration. B) Histopathologic analysis showing granulomatous inflammation with massive necrosis and small abscesses formation. Periodic acid-Schiff stain. Scale bar indicates 100 μm. C) Inclusions inside cytoplasms of foamy macrophages. Periodic acid-Schiff stain. Scale bar indicates 20 μm.

When the patient visited our hospital, a series of examinations were given. Laboratory results included a leukocyte count of 6.76 × 10^9^ cells/L (reference range 3.5 × 10^9^–9.5 × 10^9^ cells/L) with 58.3% neutrophils (reference range 50%–70%), a C-reactive protein level of 24.35 mg/L (reference value <8.0 mg/L), and an erythrocyte sedimentation rate of 89 mm/h (reference range <1–12 mm/h).

The subcutaneous nodule on the right axilla was surgically removed and tested by using pathologic examination. The result showed granulomatous inflammation with massive necrosis and small abscesses (Figure, panel B). The other subcutaneous nodules, except for the small nodules that reappeared on the waist, were then removed and tested by using pathologic examination, routine culture (aerobic and anaerobic culture for 7 days), and shotgun metagenomic sequencing (CapitalBio MedLab, https://www.bionity.com).

Pathologic examination showed granulomatous inflammation and formation of small abscesses. Routine cultures of nodules were negative, but the metagenomic sequencing result was positive for *T. whipplei* (Appendix).

To verify the result of metagenomic sequencing, we performed PCR to amplify a partial nucleotide sequence for *T. whipplei* and examined pathologic sections by using periodic acid‒Schiff stain. We sequenced the positive PCR product to further confirm the infection (Appendix). Staining results showed PAS-positive inclusions inside the cytoplasm of foamy macrophages, which was the typical pathologic feature of *T. whipplei* infection (Figure, panel C).

On the basis of the diagnosis of *T. whipplei* infection, the patient was given doxycycline (100 mg 2×/d) and hydroxychloroquine (200 mg 2×/d) orally for >1 year starting in January 2021. Six months later, the subcutaneous nodules on the waist were not palpated, and no other new subcutaneous nodules were observed. The patient remains free of relapse.

Whipple disease is a rare infectious disease. Nearly 80% of patients had arthralgia and digestive disorders before they were given a diagnosis (*3*). Skin lesions in this disease are infrequent. Erythema nodosum‒like lesions are specific manifestations in patients with *T. whipplei* infection because of the response to the immune reconstitution inflammatory reaction after initial of antimicrobial drug therapy (*4*). Our patient had multiple subcutaneous nodules and was not given any antimicrobial drugs before diagnosis. Therefore, the multiple subcutaneous nodules were considered to be primary skin lesions.

To further evaluate whether the infection affected the intestine, the patient underwent enteroscopy. Results of enteroscopy showed that there was no infection in the intestine. Symptoms was not consistent with the typical symptoms of *T. whipplei* infection and complicated the diagnosis.

Although metagenome sequencing results positive for *T. whipplei* infection are not the standard for diagnosis, this technology provided clues and improved the diagnosis. Specific PAS staining for *T. whipplei* further confirmed the result of metagenome sequencing.

On the basis of the diagnosis, we discontinued treatment with doxycycline and trimethoprim/sulfamethoxazole because of inactivity of trimethoprim and acquired resistance to sulfamethoxazole for *T. whipplei* infection (*5*). The patient was then given doxycycline and hydroxychloroquine and showed satisfactory results. However, a long-term therapeutic course and close follow-up are essential to avoid relapses and reinfection.

Whipple disease has been rarely reported in China. The few reports involved the central nervous system and respiratory system (*6*,*7*). Our report demonstrates a patient who had *T. whipplei* infection and multiple subcutaneous nodules as the initial and single symptom. Currently, treatment and follow-up are ongoing, and the therapeutic effect is satisfactory. However, lifetime susceptibility and high relapse rate pose a challenge to treatment. We hope to increase the understanding of Whipple disease through the diagnosis and treatment for this case-patient.

Appendix Additional information on subcutaneous nodule caused by *Tropheryma whipplei* infection.
