# Supplementary material for: Subcutaneous Nodules Caused by Tropheryma whipplei Infection
Source: Emerg Infect Dis. 2022 Mar;28(3):761–3. doi: 10.3201/eid2803.211989 (PMC8888228; doi:10.3201/eid2803.211989)
Supplement: Appendix — Additional information on subcutaneous nodule caused by Tropheryma whipplei infection. [file 21-1989-Techapp-s1.pdf]

Article DOI: https://doi.org/10.3201/eid2803.21-1989

Subcutaneous Nodules Caused by *Tropheryma whipp*lei Infection

Appendix

Details about Shotgun Metagenomic Sequencing

Sample Information

Sample number: B2030406_TIS

Sample type: Biopsy sample

Receipt day: December 9, 2020

Report day: December 11, 2020

Report Information

Bacterium: *Tropheryma whipplei*

Reads: 28

Total bases: 4,149 bp sequence length

3,145 bp reads

Coverage: 0.34% average depth: 1.3×

Estimated concentration: 1.1×10^1 copies/mL

PCR was performed to amplify partial sequence for *T. whipplei* and products was purified and sequenced by using first-generation sequencing technology.

Primers

TW-F: 5′-cagggaagaagcgaaagtga-3′

TW- R: 5′-accacctgtataccgacctt-3′

Tropheryma whipplei 16S rRNA (partial sequence: 1,478 bases)

1 agagtttgatcctggctcaggacgaacgctggcggcgtgcttaacacatgcaagtcgaac

61 ggaaatcagggagcttgctccctgtgattagtggcgaacgggtgagtaacacgtgagcaa

121 cctgcccctaactttgggataacttcgggaaaccggggctaataccaaatacgacccatg

181 agggcatcctctgtgggtggaaagaatttcggttggggatgggctcgcggcctatcagct

241 tgttggtggggtaacggcctaccaaggcatcgacgggtagccggcctgagagggtgatcg

301 gccacactgggactgagacacggcccagactcctacgggaggcagcagtggggaatattg

361 cacaatgggcgcaagcctgatgcagcaacgccgcgtgggggaagaaggccttcgggttgt

421 aaacctcttttggcagggaagaagcgaaagtgacggtacctgcagaaaaagcgccggcta

481 actacgtgccagcagccgcggtaatacgtagggcgcgagcgttgtccggaattattgggc

541 gtaaagagctcgtaggcggtttgtcacgtctgccgtgaaaatccgaggctcaacctcgga

601 cctgcggtgggtacgggcaggctagagtgcgataggggagagcggaattcctggtgtagc

661 ggtgaaatgcgcagatatcaggaggaacaccgatggcgaaggcagctctctgggtcgcta

721 ctgacgctgaggagcgaaagcgtggggagcgaacaggattagataccctggtagtccacg

781 ccgtaaacgttgggcgctagacgtgggggtggttccaccgcttccgtgtcgcagctaacg

841 ccattaagcgccccgcctggggagtacggccgcaaggctaaaactcaaaggaattgacggg

901 ggcccgcacaagcggcggagcatgcggattaattcaatgcaacgcgaagaaccttaccaa

961 ggcttgatatataccggaaaggcgtagagatacgccccccgcaaggtcggtatacaggtg

1021 gtgcacggttgtcgtcagctcgtgtcgtgagatgttgggttaagtcccgcaacgagcgca

1081 accctcgtcctgtgttgccagcgcgtaatggcggggactcacaggagactgccggggtca

1141 actcggaggaaggtggggatgacgtcaaatcatcatgcccttcggtcttgggcttcacgc

1201 gtgctacaatggctggtacagagggttgcaatatcgcaaggtggagcgaatctcaaaaag

1261 ccagtctcagttcggattggggtctgcaactcgaccccatgaagtcggagtcgctagtaa

1321 tcgcagatcagcaatgctgcggtgaatacgttcccgggccttgtacacaccgcccgtcaa

1381 gtcatgaaagctggtaacacccgaagccggtgacttaaccttttggagagagccgtcga

1441 aggtgggattggtgattgggactaagtcgtaacaaggt

Conclusion

Phylogenetic analysis indicated that the obtained partial sequence was from *T. whipplei*.

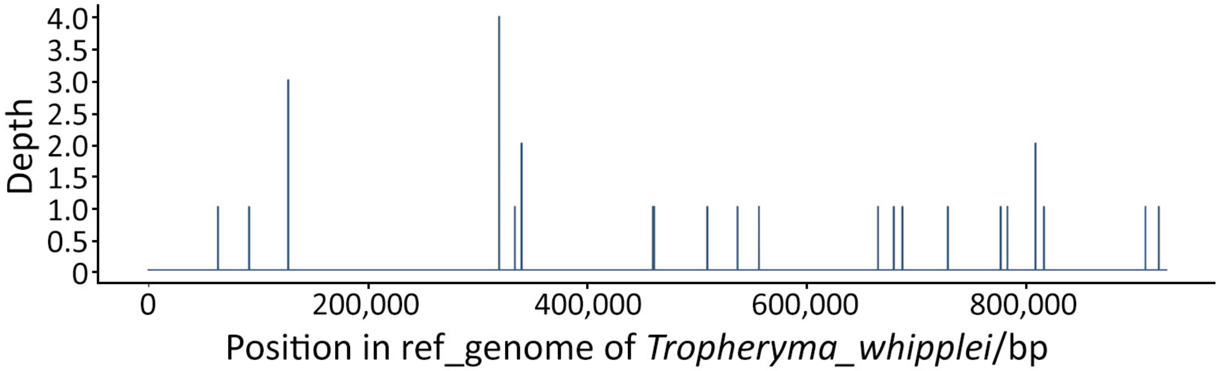

Appendix Figure 1. Mapping of *Tropheryma whipplei* reads.

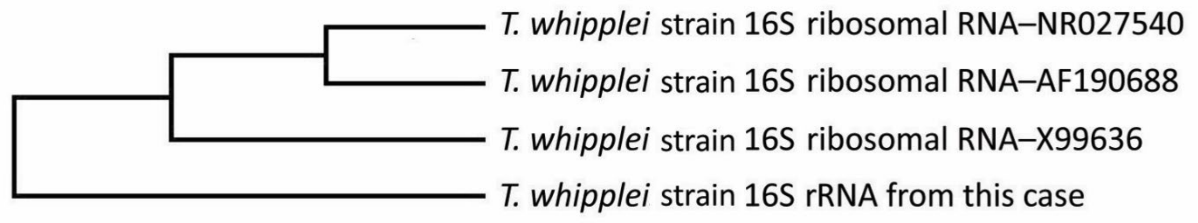

Appendix Figure 2. Phylogenetic tree of *Tropheryma whipplei* 16S rRNA sequence.
